# Nanoscale mosaicity revealed in peptide microcrystals by scanning electron nanodiffraction

**DOI:** 10.1038/s42003-018-0263-8

**Published:** 2019-01-18

**Authors:** Marcus Gallagher-Jones, Colin Ophus, Karen C. Bustillo, David R. Boyer, Ouliana Panova, Calina Glynn, Chih-Te Zee, Jim Ciston, Kevin Canton Mancia, Andrew M. Minor, Jose A. Rodriguez

**Affiliations:** 1 Department of Chemistry and Biochemistry, UCLA-DOE Institute for Genomics and Proteomics, University of California Los Angeles, Los Angeles, CA 90095 USA; 2 National Center for Electron Microscopy, Molecular Foundry, Lawrence Berkeley National Laboratory, Berkeley, CA 94720 USA; 3 Department of Materials Science and Engineering, University of California Berkeley, Berkeley, CA 94720 USA

## Abstract

Changes in lattice structure across sub-regions of protein crystals are challenging to assess when relying on whole crystal measurements. Because of this difficulty, macromolecular structure determination from protein micro and nanocrystals requires assumptions of bulk crystallinity and domain block substructure. Here we map lattice structure across micron size areas of cryogenically preserved three−dimensional peptide crystals using a nano-focused electron beam. This approach produces diffraction from as few as 1500 molecules in a crystal, is sensitive to crystal thickness and three−dimensional lattice orientation. Real-space maps reconstructed from unsupervised classification of diffraction patterns across a crystal reveal regions of crystal order/disorder and three−dimensional lattice tilts on the sub-100nm scale. The nanoscale lattice reorientation observed in the micron-sized peptide crystal lattices studied here provides a direct view of their plasticity. Knowledge of these features facilitates an improved understanding of peptide assemblies that could aid in the determination of structures from nano- and microcrystals by single or serial crystal electron diffraction.

## Abstract (web summary)

Marcus Gallagher-Jones et al. use 4DSTEM to reconstruct real-space maps of single peptide nanocrystals at cryogenic temperature. They identify regions of crystal order/disorder providing new insights into crystal mosaicity.

## Introduction

The physical and chemical properties of a crystal depend in part on its underlying lattice structure. Changes in the packing of macromolecules within crystals perturb this structure as is exemplified by crystal polymorphism^1^. Packing rearrangements can also lead to deterioration of lattice order and limit the usability of a crystal for structural determination^2,3^. Imperfections in protein crystals can in part be described by the mosaic block model^2–4^, in which monolithic crystal blocks or domains tile to form a macro-crystal, but vary in size, orientation and/or cell dimensions^3^. Because directly measuring mosaicity in protein crystals is inherently challenging^2^, crystallographic software must estimate disparities in domain block size, shape and orientation per crystal^5–7^, for full and partial Bragg reflections^5,8^. Because these domains are vastly smaller than the typical illumination in diffraction experiments, they are modelled as a continuous, but bounded, spectrum of morphology/orientation. Mosaicity varies by crystal and is affected by crystal size^9^, crystal manipulation^10^ and parameters for data collection^11^. The challenge in accurately assessing these models in protein nanocrystals has been highlighted by analysis of diffraction measured using x-ray free electron lasers^6,12^.

Direct views of a protein crystal lattice can be obtained by high-resolution electron microscopy (EM)^13,14^, facilitated by advances in high-resolution imaging^13–17^ and cryogenic sample handling techniques^13,18,19^. Cryo-EM also reveals crystal self-assembly^20–24^ and, for two-dimensional protein crystals^21^, shows natural variation between unit cells^22,23^. Domain blocks can be identified in cryo-EM images of three-dimensional (3D) lysozyme microcrystals^20^, where Fourier filtering helps estimate the location and span of multiple blocks across a single crystal^20^.

Macromolecular structures can be obtained from similar nanocrystals by selected area electron diffraction-based methods such as MicroED^25^ and rotation electron diffraction^26^. Structures determined by MicroED or similar approaches range in size from small molecules to proteins, including a variety of peptides^27–34^. In MicroED, frozen-hydrated nanocrystals are unidirectionally rotated while being illuminated by an electron beam to produce diffraction movies^35^. These movies are processed by standard crystallographic software^36^, and structures are determined and refined using electron scattering factors^37,38^. Diffraction signal permitting structures from well-ordered crystals can be determined by MicroED with atomic resolution^27,28,32^ and mirror those obtained by microfocus x-ray crystallography^33,39^. These structures represent an average over entire crystals or large crystal areas, due to the use of a selected area aperture during data collection.

In EM, greater control over illuminated areas is achieved by scanning transmission EM (STEM), which positions a focused electron beam (typically <1 nm) at discrete locations on a sample to produce images of sub-micron-thick biospecimens^40^ over large fields of view^41,42^. A variety of sample properties can be probed by collecting electrons from different angular ranges, such as annular dark field detection with low and high scattering angle detectors (ADF, HAADF)^43,44^, annular bright field detection (ABF)^45^ and differential phase contrast detection^46^, providing access to different contrast mechanisms underlying these modalities. These approaches typically rely on monolithic detectors that integrate electrons over a specific angular range originating from the sample at each probe position and attribute the signal intensity to a point on the sample^44^. These techniques have been successful in the 3D mapping of atomic features within imperfect crystals of radiation hard materials^47^.

In contrast, a scanning nanobeam diffraction experiment records diffraction patterns on a two-dimensional pixelated detector at each scan position across a sample. Each Scan_*x*_ × Scan_*y*_ position of the scan has a *K*_*x*_ × *K*_*y*_ dimension in reciprocal space (diffraction image) resulting in a four-dimensional data set (4DSTEM)^48–50^. These data can then be processed to reconstruct a real space image of the sample corresponding to specific features in the measured diffraction patterns from each scan point, resulting in a greater flexibility in the imaging contrasts obtainable from a single experiment. Cooling sensitive samples to cryogenic (liquid nitrogen) temperatures is advantageous to minimize the electron-induced radiation damage in such experiments. Using these methods, sensitive, semi-crystalline organic polymers have been investigated by 4DSTEM to reveal differential lattice orientation within thin films^41,42^.

Here we analyse beam-sensitive 3D peptide nanocrystals at liquid nitrogen temperatures by 4DSTEM. Our findings address a lack of direct estimates of nanoscale lattice variation in biomolecular microcrystals by other diffraction methodologies and address their relationship to diffraction data quality. Our measurements reveal effects that may influence MicroED experiments and other nanocrystallography methods including those performed at x-ray free electron lasers^6,12,51–54^, or synchrotron-based micro- and nanocrystallography^9,55,56^.

## Results

### 4DSTEM of 3D beam-sensitive peptide nanocrystals

**Fig. 1:**
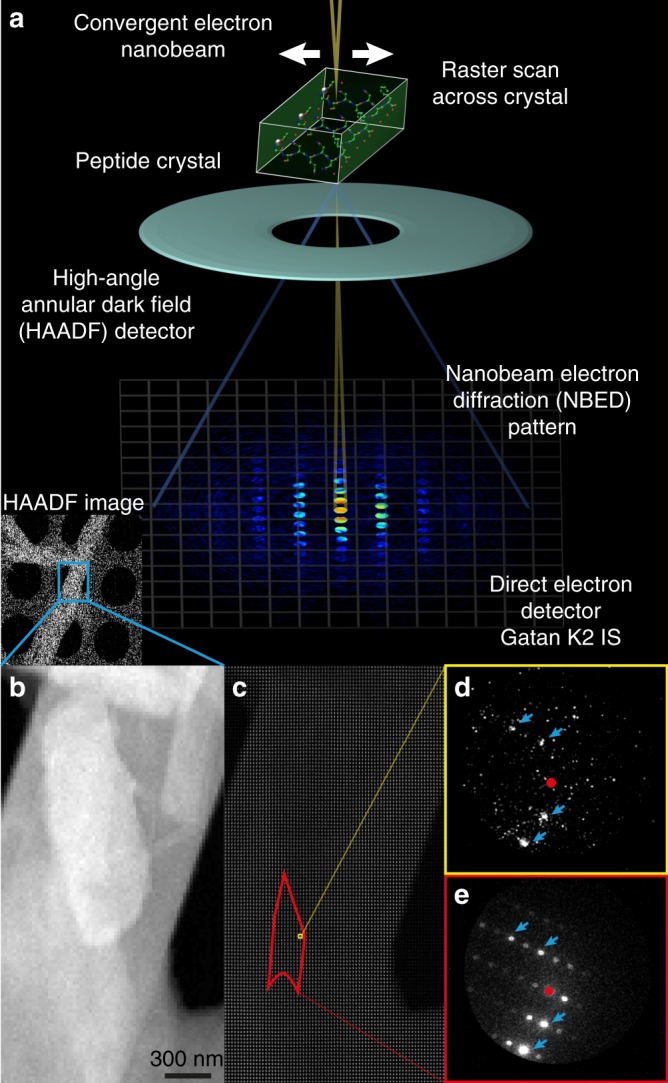
Measuring lattice structure in peptide nanocrystals by 4DSTEM. **a** Diagram of a 4DSTEM experiment shows key aspects and components of the procedure; inset shows a low dose and low magnification high-angle annular dark field (HAADF) STEM image. **b** A higher resolution STEM image shows the crystal in inset of **a** with greater detail. **c** Image montage shows all patterns collected in a 4DSTEM scan captured alongside the image in **b**; a single diffraction pattern is shown in **d**, and an average of all diffraction patterns from the red region highlighted in **c** is shown in **e**. Primary beam is masked by red circles, blue arrows indicate a subset of Bragg reflections

To assess nanoscale lattice changes within single peptide crystals, we generated two-dimensional maps of their lattice structure by 4DSTEM (Fig. 1). We evaluated 34 nanocrystals formed by a prion peptide segment with sequence QYNNQNNFV, whose structure has been previously determined (PDB ID 6AXZ) by MicroED to sub-Å resolution^33^. Crystals analysed by 4DSTEM were equivalent in shape and size to those evaluated by MicroED; most were needle shaped and several microns in their longest dimension, but less than a micron thick and wide (Supplementary Figure 1). Crystals that lay over holes on the quantifoil^®^ grid were found to produce the highest contrast signal and were chosen for analysis by 4DSTEM.

During 4DSTEM data collection, an approximately six nanometre electron beam (Supplementary Figure 2) was scanned coarsely across a grid, while regions of interest were identified by low-mag, low-dose STEM imaging. Fine scans were performed on up to 3-μm-long regions of single nanocrystals, while diffraction patterns were measured at each step using a direct electron detector (Fig. 1c). Beam parameters in 4DSTEM scans were comparable to those of prior studies (Supplementary Table 1), with an estimated fluence of ~1 e^−^ Å^−2^ in regions of the crystal exposed to the beam. For all 4DSTEM scan points, we applied the lowest dose necessary to detect Bragg reflections (Fig. 1d, e, Supplementary Figure 1). We confirmed that the dose was sufficiently low by measuring several overlapping scans from the same area of a single crystal and found that decay in the highest resolution reflections occurred only after multiple scans of the same area (Supplementary Figure 3). The probe dwell time and diffraction image exposure were synchronized and produced full-frame patterns at a rate of 400 s^−1^ (Supplementary Figure 4).

These parameters yielded diffraction patterns whose resolution extended beyond 1.4 Å (Supplementary Figure 1), measured from only an estimated 1000–15,000 diffracting molecules given an effective illumination area of 6 × 6 nm, a crystal thickness of 100–500 nm and unit cell constants of *a* = 4.94, *b* = 10.34, *c* = 31.15, *α* = 94.21, *β* = 92.36, *γ* = 102.2. Bragg diffraction was observed only from within the bounds of the crystals, as identified by annular dark field STEM images (Fig. 1, Supplementary Figure 1, 3). Diffraction patterns integrated over micron-sized regions of single crystals matched MicroED patterns measured from a micron-sized selected area (Supplementary Figure 1).

### Diffraction pattern reconstruction by hybrid electron counting

Conventional electron counting in HRTEM is achieved by thresholding images acquired using a fast direct electron detector based on estimates of detector readout and Landau noise^17^. Coincidence loss is minimized by operating the detector at a sufficiently high frame rate and low electron dose^17^. This procedure limits the dynamic range in diffraction images because coincidence loss is difficult to escape at Bragg reflections, where signal is concentrated compared to background regions (Supplementary Figure 4). This phenomenon is exacerbated most by detection of the focused central beam, where coincidence loss is high (Supplementary Figure 5). To benefit from the improved signal to noise afforded by electron counting, while maintaining some of the dynamic range lost due to coincidence, we implemented a hybrid method for signal detection (Supplementary Figure 5). This procedure introduced little change in the signal observed at most Bragg reflections compared to standard counting, but improved estimates of central beam intensity (Supplementary Figure 5).

Raw patterns collected on a K2 direct electron detector at 400 frames s^−1^ were processed using a workflow that involves background subtraction, normalization, thresholding, and pixel-level stepwise counting (Supplementary Figure 4). The hybrid counting approach was implemented as follows: We used the distribution of pixel intensities within an entire 4DSTEM scan to estimate background signal and categorize measured pixel intensities into counting bins corresponding to single or multiple electron counts (Supplementary Figure 4e, 5a). Based on this criterion, the majority of pixels in a pattern were zero valued (Supplementary Figure 4e); a small fraction (<1%) within the regions where Bragg reflections were expected received single counts and a lesser minority of pixels were assigned multiple counts corresponding to measurement of coincident electrons (Supplementary Figure 5a). Of the latter group, most events occurred within the region illuminated by the central (000) focused beam disc (Supplementary Figure 5a (insets)), particularly over thin sample regions or holes in the carbon support (Supplementary Figure 5b, c).

The range of signal counts in processed patterns spanned values from 1 to 35, but very few pixels were assigned values >10 (Supplementary Figure 5a). While the true degree of coincidence cannot be measured by this method, comparison of these patterns to those processed with a binary threshold shows an increase in dynamic range achieved by hybrid counting protocols (Supplementary Figure 4f, 5b, c). This process resulted in an estimate of 2612 ± 445 electrons per pattern, with 222 ± 146 electrons being localized to Bragg reflections. A small fraction of electrons that are uncounted scatter beyond the view of the pixelated detector and are detected by the HAADF. These measurements approximate the 3750 electrons expected to impinge upon a crystal region of this size.

Pixel-wise processing of data also allowed us to correct diffraction pattern shifts due to beam scanning using centre of mass alignment (Supplementary Figure 6). The majority of scans presented minimal shifts; some scans showed preferential drift of the diffraction pattern along a single orientation (Supplementary Figure 6c, d (inset)). The combined intensity of the central beam and Bragg reflections allowed us to estimate crystal thickness at each scan point (Supplementary Figure 7). This estimated thickness correlates well with features observed in annular dark field STEM images including empty regions within holes and the carbon support (Supplementary Figure 7).

### Analysis of lattice structure in 4DSTEM scans across large areas of peptide crystals

**Fig. 2:**
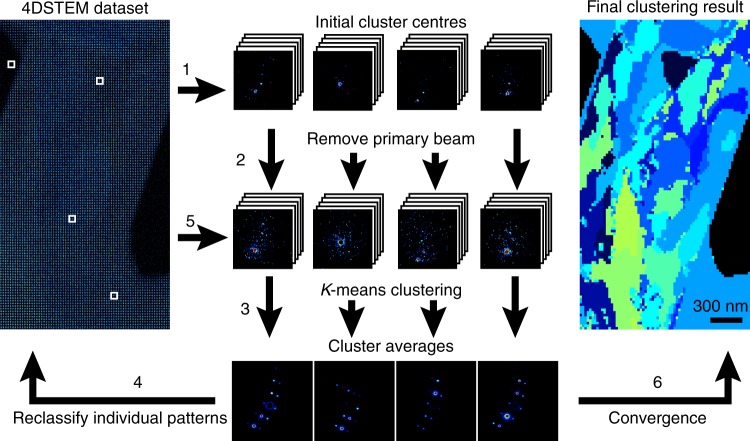
Workflow of unsupervised clustering used to define regions of similar diffraction in peptide crystals. (Step 1) Initial cluster 'centres' are assigned within the 4DSTEM dataset (left white boxes) and all patterns are binned. (Step 2) The primary beam is subsequently masked to prevent its influence on clustering. (Step 3) Individual patterns are then compared to each cluster centre sequentially via Euclidean distance and assigned to the cluster where this distance is smallest. (Step 4) Average patterns are calculated for each cluster and (step 5) are used to reclassify individual patterns until convergence (step 6) where a final map illustrating spatial localization of similar diffraction patterns is produced

To better understand changes in the pattern of Bragg reflections observed across different areas of a single crystal, we performed unsupervised classification of patterns in each scan (Fig. 2). Given the high sparsity of signal outside the central disk in individual diffraction patterns (Supplementary Figure 4, 5), we masked out the central beam to limit its influence on pattern classification (Fig. 2). This procedure revealed groups of patterns within a scan that shared a common lattice; patterns could be classified in this way for all measured crystals. The number of regions identified by classification varied between crystals and ranged from 4 to 20 with an average area of 1.6 × 10^5^ ± 1.7 × 10^5^ nm^2^ (Supplementary Figure 8). In 2 of the 34 datasets analysed, the number of clusters was manually assigned because fewer than five clusters were reproducibly identified. The spatial distribution of clusters identified by this approach was consistent with changes in the pattern of Bragg reflections across a scan, rather than other background signal or other diffuse scattering (Supplementary Figure 9).

**Fig. 3:**
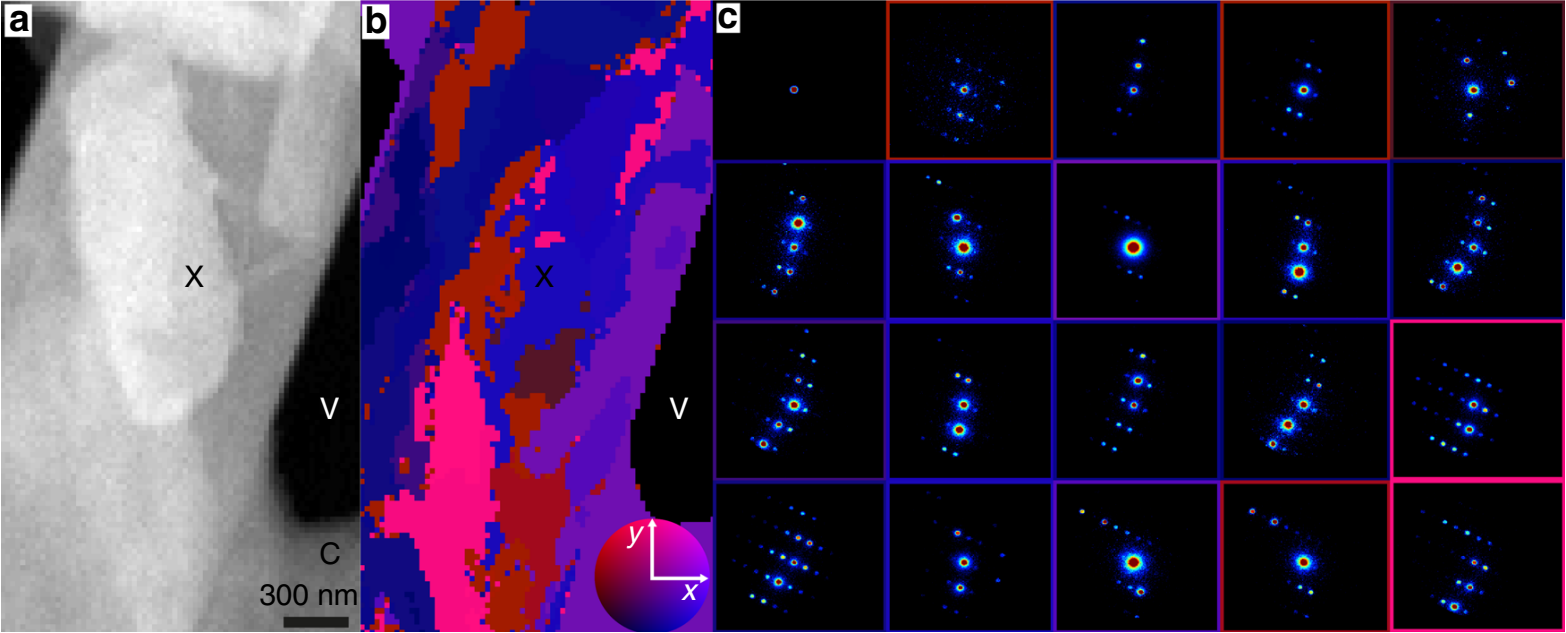
Mapping of nanoscale lattice reorientation within peptide nanocrystals by 4DSTEM. **a** HAADF image of a QYNNQNNFV nanocrystal. Unsupervised classification of diffraction patterns captured by the 4DSTEM scan acquired during the measurement of **a** are shown in **b**; this is a map of diffraction clusters not obvious from **a**. Colours in **b** illustrate the change in lattice orientation for each individual cluster with respect to the mean orientation across the scan area. The colour wheel (inset) demonstrates the relative orientation away from the mean in *x* and *y* tilts; maximum deviation denoted by the colour wheel is 4°. Average diffraction patterns from diffraction outlined in **b** are shown in **c**, where the colour of the bounding box corresponds to the colour of the corresponding cluster in **b**. C, X and V indicate carbon support, peptide crystal and vacuum, respectively

The number and intensity of Bragg reflections varied across classified patterns (Fig. 3c, Supplementary Figure 10). Bragg reflections were weak or attenuated in regions where crystals appeared thickest and in thin regions where the central beam showed high counts (Fig. 3c, Supplementary Figure 10). The overall pattern of Bragg reflections differed across a single crystal, changing in a coordinated fashion at all resolutions. Diffraction patterns in a cluster appeared spatially linked within a crystal (Fig. 3b). This effect was reinforced by the requirement that patterns be assigned to a particular class. When mapped onto crystals, diffraction pattern clusters gave the appearance of nanodomains with definite boundaries (Fig. 3a, Supplementary Figure 8b). However, these boundaries were deceptive, since in reality the change in diffraction appeared more continuous (Supplementary Figure 10). To assess the true extent of angular reorientation, we performed a library-based indexing^57^ of patterns from four crystals obtained by cluster averaging (Supplementary Figure 11). These particular crystals were selected as they contained the necessary number of reflections per cluster to unambiguously assign a lattice orientation. Library indexing of patterns from these four crystals showed that changes in diffraction across clusters could be attributed to an average ±1° tilt of the lattice away from the mean orientation of the crystal (Fig. 3c). The minimum deviation observed in the analysed crystals was ±0.5°, while the maximum deviation was ±4°. While the degree of variation in lattice tilt differs between crystals, lattice tilt was observed in all 34 crystals investigated in this study.

### Analysis of lattice structure in HRTEM images of peptide crystals

To obtain context for the spatial distribution of changes in lattice structure in peptide crystals we observed by 4DSTEM, we performed HRTEM of similar crystals in search of similar effects^20^. High-resolution cryo-EM on crystals of QYNNQNNFV with a total dose of 27.8 e^−^ Å^−2^ per image (Supplementary Figure 12a, c) allowed us to directly visualize the lattice in these crystals at approximately 2.3 Å resolution (Supplementary Figure 12b, d). We processed these images by single-particle cryo-EM protocols, identifying image sub-regions for unsupervised clustering, analogous to the procedure used for 4DSTEM pattern classification (Supplementary Figure 13).

Fourier transforms of whole crystal images showed clear variation in Bragg reflections when different crystallites were imaged or when comparing crystalline features to empty regions within holes or regions of carbon support (Supplementary Figure 12, 13). Cluster averages also showed differing images (Figure 13c, f, Supplementary Figure 14a-d), but quantifiable differences between clusters were difficult to detect in either real or reciprocal space (Supplementary Figure 14a-h). This is potentially due to the influence of crystal thickness and defocus across the image that make true changes in 3D lattice character difficult to discern (Supplementary Figure 13).

## Discussion

To reveal the lattice substructure within beam-sensitive 3D crystals, we performed 4DSTEM on peptide nanocrystals at cryogenic temperatures. In contrast to semi-crystalline polymers studied previously by 4DSTEM^41,42^, the prion peptide nanocrystals we evaluated are composed of highly ordered peptide arrays that diffract to sub-ångstrom resolution^33^. Taking advantage of the known lattice parameters for these crystals, their highly ordered structure and the known atomic arrangement of their constituent molecules, we probed for nano-scale changes in diffraction across single crystals. The data we obtained add to previous studies on the lattice substructure and physical properties of nanometre- to micrometre-sized protein crystals investigated by a variety of methods^6,20,25,26,58^.

Unlike other methods of protein crystal characterization^20,59–61^, 4DSTEM provides direct observation of lattice character through nanoscale mapping of changes in diffraction across micron-scale areas^41^. Our measurement of 1.4 Å resolution diffraction from sub-10 nm regions of peptide crystals was facilitated by three key technological features of our experiment: fast readout direct electron counting detectors^62^, a hybrid counting protocol applied to sparse diffraction data captured by 4DSTEM and low-dose cryogenic techniques that lessen the evidence of radiation damage. Because of these key features, the diffracted resolution we achieve in 4DSTEM scans is comparable to that of diffraction patterns measured by MicroED using a selected area aperture. The fluence required to achieve this resolution by 4DSTEM is higher than it is for MicroED, on the order of 1 e^−^ Å^−2^ for the former compared to about 0.01 e^−^ Å^−2^ for the latter^63^. The dose chosen for these 4DSTEM experiments is necessary to achieve high-resolution diffraction from a lower number of molecules diffracted at each scan point (~1–10 × 10^3^ molecules) compared to those diffracted by MicroED from similar crystals (~1 × 10^7^ molecules), even for the smallest selected area apertures^27,35^. The higher potential for damage by this dose at each scan position is mitigated by spacing scan steps by a distance larger than the probe size, ensuring that an unprobed region of the crystal is illuminated with each scan step. While several step sizes were explored across scans, a step size of 20 nm was sufficiently fine to allow pattern classification yet large enough to avoid perceptible pattern degradation due to damage from neighboring regions.

The changes in diffraction we observe across micron-sized regions of peptide nanocrystals point to inhomogeneities across a single crystal. These inhomogeneities did not necessarily correspond to those observed in HAADF images alone, as it appears that only sub-regions of crystals produce strong, measurable diffraction. Differences in diffraction are not only diagnostic of regions with strong and weak diffraction within individual crystals but also point to changes in the orientation of the lattice within a crystal. This nanoscale reorientation in sub-micron regions of a single nanocrystal is difficult to detect by methods that make use of selected area electron diffraction, as well as those that integrate diffraction from whole crystals. This is especially true after data from multiple crystals is merged^64,65^, a requirement for both serial crystallography and most MicroED experiments^35^. The real space resolution of a map produced by 4DSTEM with a step size of 20 nm is contrasted with the real space size of the selected area aperture in MicroED of 500–1000 nm, allowing much finer sampling of lattice variation. Lattice reorientation at this scale in macromolecular nanocrystals may explain the discrepancy in dynamical scattering observed from crystals of this thickness by MicroED, compared to what would be expected by simulation from perfect crystals^66^.

The nanoscale lattice reorientation observed by 4DSTEM differs from conventional domain blocks. Whereas conventional mosaic blocks have been modelled as a continuous, semi-random distribution of orientations, we observe a gradual, progressive change in orientation as a function of spatial localization. Presently, our library-based orientation assignment acts on the average diffraction over clustered patterns and requires known cell constants, but improved interpretation of single sparse patterns and ab initio indexing of electron diffraction patterns may alleviate one or both of these limitations. Despite present limitations, nano-scale lattice changes in our crystals are more readily identifiable from 4DSTEM scans than from HRTEM images of similar nanocrystals owing to the higher contrast of Bragg peaks captured by diffraction. Transforms of sub-regions within a 3D lysozyme nanocrystal have shown similar lattice differences across a single crystal^20^. However, in that case, the changes were also potentially attributable to differences in defocus across the crystal or to changes in the level of dynamical scattering across non-uniformly thick areas of the crystal^20^. Interpretation of our own images of frozen-hydrated peptide nanocrystals presents similar challenges (Supplementary Figure 12, 13). 4DSTEM allows a quantitative classification of lattice orientations within a crystal and can reveal more subtle changes than those identified in HRTEM images of similar crystals. This difference may point to a greater sensitivity to detection of lattice changes by 4DSTEM at even lower doses than those required for HRTEM.

The lattice tilts we identify may ultimately represent the result of various physical phenomena: they may be intrinsic properties of the crystals themselves, limited to only a subset of macromolecular crystals and affected by or result from sample preparation procedures. Current methods in cryoEM sample preparation, for example, may exert forces potent enough to distort crystal lattices and affect molecular structures^67^. Lastly, by allowing nanoscale (20 nm) interrogation of lattice structure in macromolecular crystals, 4DSTEM offers a potential way to shrink the number of molecules required for structure determination by electron diffraction from single or serial crystal data.

## Methods

### Preparation of crystals

Crystals were grown as previously described^33^. Briefly, synthetic peptide (GenScript) with sequence QYNNQNNFV at a purity of 98% was dissolved in ultrapure water to a final concentration of 3.5 mM. QYNNQNNFV crystals were formed in batch by mixing dissolved peptide solution 1:1 with a buffer solution composed of 10% MPD ((*RS*)-2-methyl-2,4-pentanediol) and 0.1 M MES (2-(*N*-morpholino)ethanesulfonic acid) at pH 6.0.

### 4DSTEM data collection strategy

Crystal suspensions were diluted two-fold in water or crystallization buffer before being dispensed onto holey carbon grids (Quantifoil 2/4, #300 copper; Ted Pella Inc.) and allowed to air dry. Samples were introduced into the TEAM I microscope (FEI Titan) with a Gatan 636 Cryo holder, and cooled to liquid nitrogen temperatures throughout all data acquisition. The TEAM I microscope was operated at 300 keV in STEM mode with a convergence half-angle of 0.5 mrad resulting in a ~6 nm convergent beam (Supplementary Figure 2).

Crystals of interest were identified using annular dark-field STEM at low magnification (Fig. 1a (inset)). For this, the electron fluence (dose, e^− ^Å^−2^) was limited to a fluence of < 0.01 e^−^ Å^−2^ to minimize diffraction decay due to radiation damage, starting with parameters identified for other beam-sensitive organic lattices^41^ (Supplementary Table 1). The search for crystals was performed by scanning a focused beam with a dwell time of 1 µs over a 512 × 512 scans across 14 × 14 μm fields of view, corresponding to an electron fluence < 0.01 e^−^ A^−2^. We chose single crystals or crystal bundles visible at low magnification in annular dark field STEM images for further evaluation by 4DSTEM (Fig. 1a–c).

4DSTEM datasets were collected by scanning the 6 nm probe over the sample in two-dimensional scan using a 2.5 ms per step dwell time and a 20 nm step size. The electrostatic lens above the probe-forming aperture was adjusted such that the overall fluence per scan step was ~1 e^−^ Å^−2^. Diffraction patterns were recorded using a Gatan K2-IS direct electron detector (GATAN), with a 1792 × 1920 pixel detection and an effective camera length of 575 mm (Fig. 1a). Diffraction in 4DSTEM patterns was minimally occluded by the angular dark field detector. Typical scans consisted of 1000 to 10,000 diffraction patterns captured at 400 frames s^−1^; datasets were ~5–30 GB in size.

### 4DSTEM dose estimation

The total dose imparted per 4DSTEM scan step was estimated as follows: The convergent probe was imaged in TEM mode at high magnification using a Gatan US1000 CCD detector with an exposure time of 10 s and all other beam settings the same as those used for data acquisition (Supplementary Figure 2). Using a nominal conversion rate of 3 counts per e^−^ at 300 keV, we estimated the total number of electrons within the probe to be 1.5 × 10^6^ e^−^ s^−1^. This number calculated from the image of the probe agrees with the screen current estimate for a similar gun lens setting. The diameter of the probe at FWHM was measured to be 6 nm (Supplementary Figure 2) and, given an exposure time of 0.0025 s, the final dose per scan step was estimated as ~1 e^−^ Å^−2^. The calculated dose above is different than that calculated from the field of view which would have been reported as an order of magnitude less due to the real space ‘pixel size’ of 15–20 nm.

### Image processing and hybrid counting of 4DSTEM data

All data processing was performed using custom scripts written in the MATLAB (MathWorks) programming language. Patterns belonging to a single area scan were jointly processed to achieve hybrid counting as follows. We computed an average pattern from all of the images within each dataset (Supplementary Figure 4). We estimated a differential dark current offset between detector strips as the median value of each strip of pixels in the vertical direction of the measured patterns (Supplementary Figure 4c). These values were subsequently subtracted from each diffraction pattern within the dataset (Supplementary Figure 4d). After dark current correction, a Gaussian background was fit to the distribution of background subtracted pixel values of all patterns within the dataset (Supplementary Figure 4e). This fit was used to estimate a threshold for the detector dark noise such that counts that were below 5 SD of the Gaussian fit were considered background (Supplementary Figure 4e). This threshold would typically be used in standard electron counting algorithms^16,17,68^.

To recover some of the dynamic range lost due to coincident electron events we implemented a ‘hybrid counting’ approach. Here we divided all pixel values by the calculated threshold and the floor function of these values was taken to give an estimate of the degree of coincident electron events occurring at individual pixels on the detector. (Supplementary Figure 5). We found that correcting in this way resulted in the majority of events being either single or double counts and reduced the noise floor to close to zero, crucial for accurate clustering. However, in some cases where there was direct transmission of the electron beam, where the scan passed over vacuum, counts were much higher (Supplementary Figure 5 (inset)). While the assumption of a linear relationship between the detector counts and electron coincidence is incorrect, this method better captured the true transmission of the central beam than considering all counts above the threshold as single electron events. This was important for more accurately estimating crystal thickness. Finally, zero values were discarded and the 2D images were converted to coordinate lists and corresponding electron counts, an ~600-fold reduction in data size. We note that our choice of detector does not preclude its execution with other types of detector systems. Scintillator-based CMOS (complementary metal-oxide semiconductor) cameras are used routinely for selected area electron diffraction and could be used for 4DSTEM experiments, while hybrid pixel detectors offer similar electron sensitivity to the K2 used in these experiments, but benefit from a much higher dynamic range; for the hybrid pixel detectors, electron counting corrections would not be necessary^69^.

### Correction of diffraction shift in 4DSTEM scans

The horizontal and vertical shift of diffraction patterns induced by beam tilt during these relatively low magnification scans was corrected by tracking the centre of mass of the transmitted beam. As the signal to noise in a single pattern was not sufficient to calculate this accurately, we separated the problem into two steps, independently correcting shifts in the *x*-scan and *y*-scan directions. A strip-wise average of patterns was taken along the direction to be corrected; for example, for a 72 × 50 scan we would first average patterns along the first dimension to give 1 × 50 strip-wise averaged patterns. The centre of mass of the transmitted beam was used to give an estimate of the pixel shift for each strip and individual datasets within this strip were shifted to a common centre based on this. This process was repeated in the second dimension. For most datasets, just one round of shift correction was sufficient, but in cases where the shift was particularly problematic, several rounds were necessary (Supplementary Figure 6).

### Estimation of crystal thickness

Crystal thickness was estimated using the log-ratio formula typically employed in EELS (electron energy loss spectroscopy) for inelastic scattering:

$$Z_{xy} = - \lambda \cdot \ln \left( {\frac{{I_{xy}}}{{I_0}}} \right),$$

where *Z*_*xy*_ is the thickness of the crystal at scan location *xy* and *λ* is the mean free path of electrons through the peptide crystals. A *λ*-value of 332 nm was used based on estimates previously determined from equivalent crystals^70^. *I*_*xy*_ is the integrated transmitted beam at scan position *xy* (Supplementary Figure 7a) plus the integrated intensity at all Bragg peak positions (Supplementary Figure 7b). This sum represents intensity variation due to changes in inelastic scattering and was used for thickness estimates (Supplementary Figure 7g). *I*_0_ is calculated as the mean of the integrated central beam at scan positions that were over vacuum minus two times the standard deviation of values in this region. This correction was to account for fluctuations observed in the central beam intensity.

### HRTEM data collection and image processing

A monodisperse solution of QYNNQNNFV crystals was applied onto holey carbon grids (Quantifoil 1/4, #300 copper; Ted Pella Inc.) and plunge-frozen using a Vitrobot Mark IV robot (Thermo Fisher Scientific). Data were collected on a CS aberration corrected FEI Titan Krios (Thermo Fisher Scientific) operated at 300KeV. Super-resolution movies were recorded using a Gatan K2 Summit direct electron detector. The nominal physical pixel size was 1.04 Å per pixel (0.52 Å per pixel in super-resolution movie frames) and dose per frame was 1.39 e^−^Å^−2^. A total of 20 frames were taken for each movie resulting in a final dose of 27.8 e^−^Å^−2^ per image.

Super-resolution movie stacks were corrected for gain- and beam-induced motion using MotionCorr2^71^. Initial estimates of anisotropic magnification were made using 10 micrographs where crystalline ice was present using the program mag-distortion-estimate^72^. These parameters were used to correct for anisotropic magnification in MotionCorr2. Frames were subsequently aligned without using patches, dose-weighted, and down-sampled by two.

### Lattice mapping in 4DSTEM and HRTEM

Before lattice mapping individual diffraction patterns were binned 8-fold to increase their SNR. The central beam was then masked out to remove the influence of transmission from the lattice mapping; we noticed that without this step the lattice maps produced matched variations in the thickness of the crystal too closely. We used *k*-means clustering to sort diffraction patterns from a single 4DSTEM dataset into different clusters based on their Euclidian distance from the average pattern within a particular cluster:

$$\mathop {{{\mathrm{argmin}}}}\limits_s \mathop {\sum }\limits_{i = 1}^K \mathop {\sum }\limits_{x \in S_i} ||x - \mu {i^2}||,$$

where *K* is the number of clusters determined using an implementation of the *G*-means algorithm with *a* = 0.001^73^. *S*_*i*_ is an individual cluster, *µ*_*I*_ is the current average of all patterns within the cluster *S*_*i*_ and *x* is an individual diffraction pattern within the 4DSTEM dataset. The *k*-means++ algorithm was used to initialize *µ*_*i*_ for each cluster^74^. The algorithm was stopped when either 100 iterations were performed or when the within-cluster sum of squares score stagnated. Clusters where the beam passed over vacuum showed little signal when the primary beam was excluded, preventing distances between patterns in this cluster from showing Gaussian behaviour; their Euclidean distances from the mean would be close to unity. To circumvent this, we included a break in our implementation of *G*-means that stopped assignment of clusters once at least 80% of the assigned clusters were found to be Gaussian by the Anderson–Darling statistic. This substantially improved convergence of the algorithm.

For real space images collected by HRTEM, a similar procedure was followed with the following modifications. First 128 × 128 sub-images were cropped from the larger HRTEM images with no overlapping regions. We estimated *K* by the elbow method^75^, which we found to be more stable than the Anderson–Darling statistic for the noisier imaging data. The clustering step was applied as above, with the inclusion of a 5 × 5 pixel-wise search to account for potential misalignments between the two images under consideration.

### Indexing of lattices in 4DSTEM cluster averages

To assess the underlying lattice reorganization that could account for the changes in diffraction observed from the clustering, we indexed the cluster averages via library matching^75^ (Supplementary Figure 11). A library of nano-beam electron diffraction (NBED) patterns were simulated with PRISM^76^ using the known crystal structure of QYNNQNFV. We simulated expected NBED patterns arising from a 40 × 40 nm region of a perfect crystal of varying thickness (10–600 nm). Probe size and convergence semi-angle were fixed to match experimental parameters and patterns were calculated at a range of *xy* tilts, ±4° in 0.25° increments, away from the *hk*0 zone or mean orientation in cases where the crystal was not sitting close to a zone axis.

To match the experimental patterns to the simulated library, the positions of all possible peaks that could arise within the bounds of the HAADF detector were identified, excluding the central disk. These peak locations were then used to create a list of intensities by integrating all pixel values within a 4-pixel radius centred around each peaks *kxky* position. For each cluster average and simulated pattern, the listed intensities were scaled to be a ratio of the most intense peak within that pattern. Intensity lists were then compared by root-mean-square deviation (RMSD) of intensity values at all peak positions within a given pair of patterns such that:

$${\mathrm{RMSD}}_{ij} = \sqrt {\frac{{\mathop {\sum }\nolimits_{p = 1}^P \left( {{\mathrm{\mu }}i_p - {\rm Sim}j_p} \right)^2}}{P}},$$

where *p* represents an individual peak position from the set of peak positions *P*, *µi* is the *i*th averaged cluster pattern and Sim*j* is the *j*th pattern within the simulated library. The orientation of the simulated pattern with the lowest RMSD to the current cluster average was then assigned to the orientation of the lattice within that region.

### Fourier filtering of HRTEM images

To improve the contrast of the average lattice images captured by HRTEM, Fourier filtering was performed in a similar manner to Erickson and Klug^77^. For each class average the Fourier transform was computed and the mean and standard deviation were calculated for the magnitude of all pixel values, excluding the DC term. All pixels in the Fourier transform with a magnitude lower than 2 SD above the mean magnitude were set to zero, and the final filtered image was calculated by inverse Fourier transform.

### Code availability

The MATLAB scripts for data pre-processing, clustering, orientation assignment can be found at: https://github.com/marcusgj13/4DSTEM_dataAnalysis.

## Supplementary Information

Supplementary Information

## Data Availability

The pre-processed data used for clustering and orientation assignment are available at https://github.com/marcusgj13/4DSTEM_dataAnalysis. The associated raw.dm4 files are available from the EMPIAR data base at EMBL-EBI (EMPIAR-10231)
