# Supplementary material for: Nanoscale mosaicity revealed in peptide microcrystals by scanning electron nanodiffraction
Source: Commun Biol. 2019 Jan 18;2:26. doi: 10.1038/s42003-018-0263-8 (PMC6338664; doi:10.1038/s42003-018-0263-8)
Supplement: Supplementary file 1 — Supplementary Information [file 42003_2018_263_MOESM1_ESM.pdf]

**Supplementary Figures**

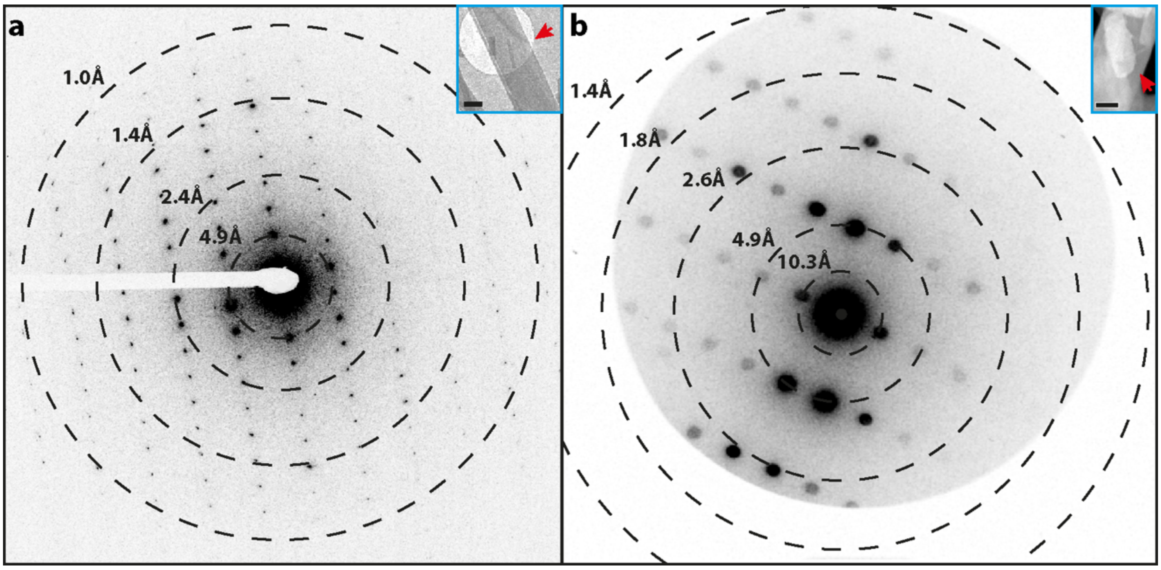


Supplementary Figure 1. **Comparison of diffraction patterns collected by MicroED and 4DSTEM.** (**a**) A diffraction photograph measured by continuous rotation MicroED from a frozen hydrated crystal of QYNNQNNFV (inset). (**b**) Diffraction pattern calculated by taking the average of all patterns within a single 4DSTEM dataset acquired from a QYNNQNNFV crystal (inset) from the same batch as (**a**). Scale bars are 300 nm in all images.

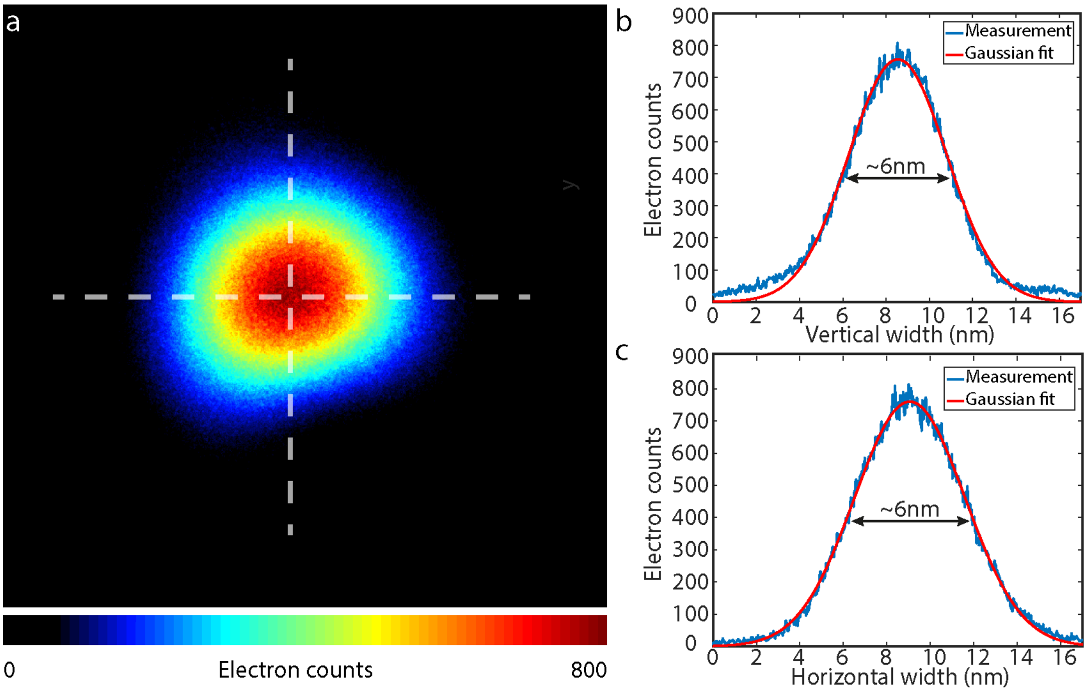


Supplementary Figure 2. **Measure of probe properties used for 4DSTEM.** (a) Image of the probe captured on a Gatan Ultra scan 1000 with an exposure time of 10s. (b) Vertical line scan across the probe (white dashed line in (a)) and Gaussian fit to the profile giving a diameter of ~6nm at FWHM. (c) Horizontal line scan across the probe (white dashed line in (a)) and Gaussian fit to the profile giving a diameter of ~6nm at FWHM.

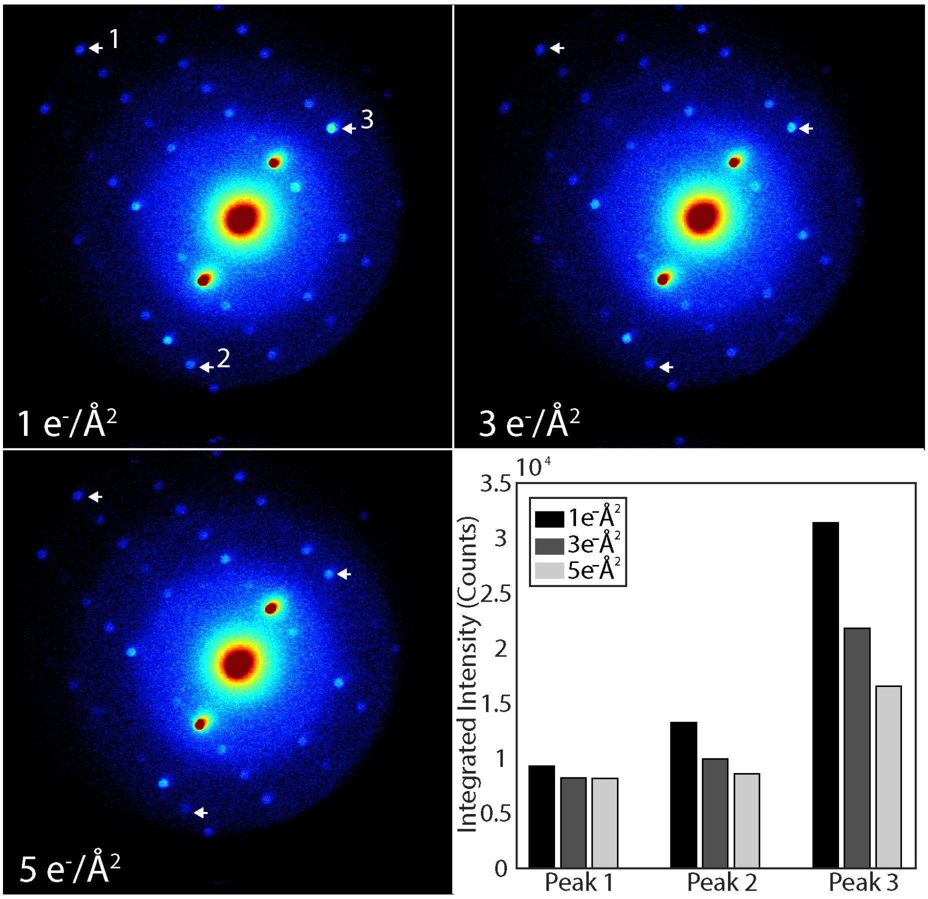


Supplementary Figure 3. **Changes in Bragg reflection brightness as a function of electron dose in 4DSTEM experiments.** (Top left to bottom left) Average diffraction patterns of 4DSTEM datasets successively acquired from the same crystal (top left: first pass, top right: third pass, bottom left: fifth pass) with the total accumulated dose below. White arrow indicates high resolution Bragg reflections whose intensity decreases as a function of dose. (Bottom right) Bar chart showing the intensity decay of individual reflections as a function of dose (as labelled in top left).

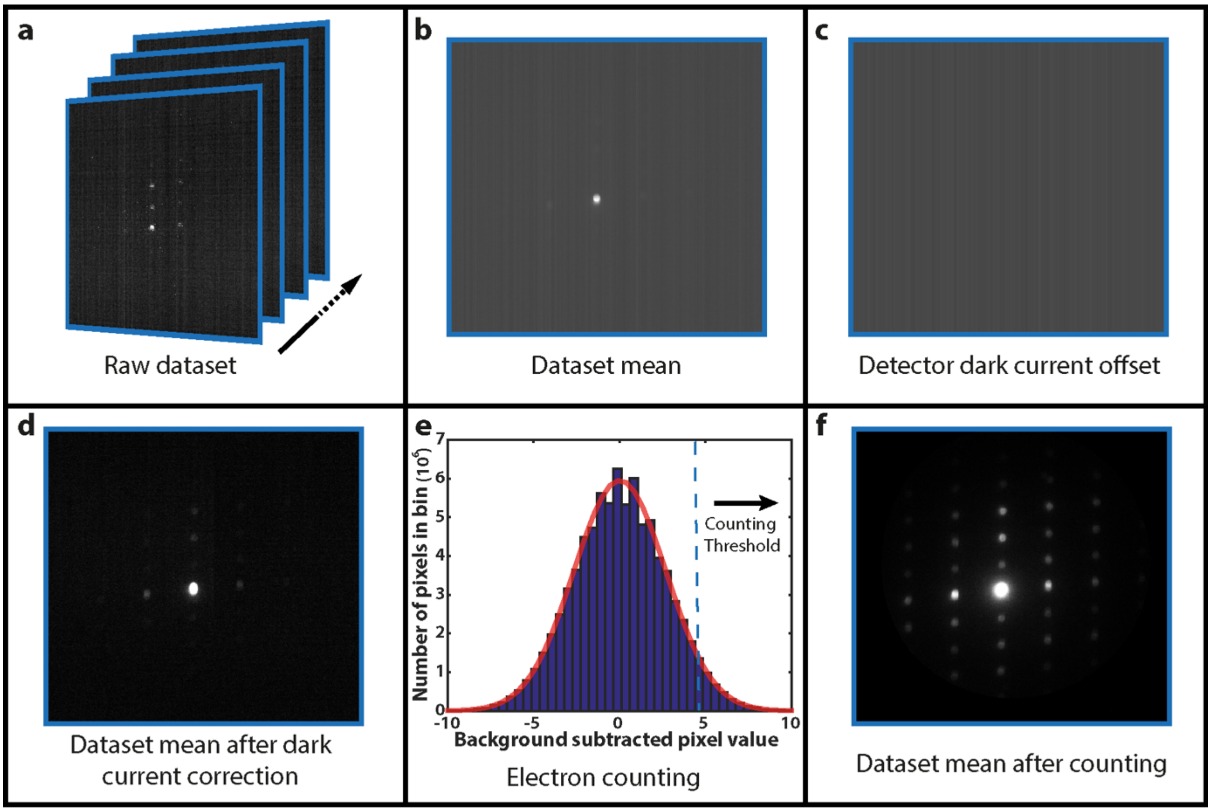


Supplementary Figure 4. **Data processing pipeline for 4DSTEM data.** (a) Raw diffraction patterns recorded at 400 frames per second are first combined to obtain a mean pattern (b). A dark current offset across the detector is estimated from the mean pattern using a median filter (c) and subsequently subtracted from all images (d). A Gaussian background is then fit to the distribution of all pixel values within the background corrected data and based on this a threshold is determined above which the incidence of electrons is considered true (e); intensity values are then converted to electron counts (f).

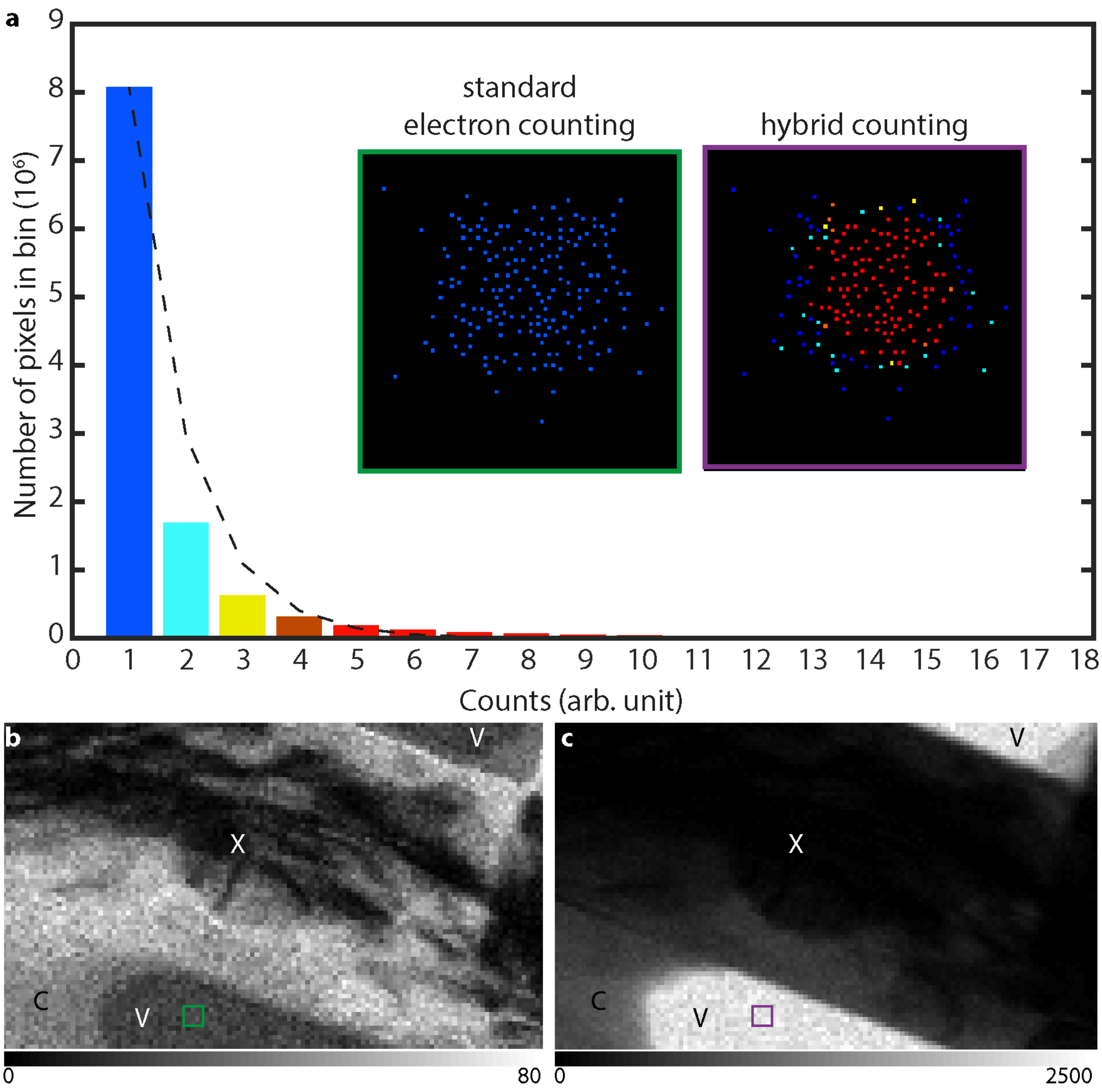


Supplementary Figure 5. **Use of hybrid counting to improve estimates of beam transmission.** (**a**) Histogram of the distribution of counts due to individual and multiple electron events recorded in a representative 4DSTEM dataset after hybrid counting. (inset) Image of the primary beam over vacuum using standard binary electron counting (left) and hybrid counting (right) from the green and purple square region in (**b**) and (**c**) respectively. Colours in insets match the colours in the histogram (images are truncated at 5 counts to improve visibility of low counts). The dashed line shows the best fit of the count histogram to a Poisson distribution. (**b**) Map of central beam intensity using standard electron counting. (**c**) Map of central beam intensity using hybrid counting. C, X and V represent areas over carbon support, peptide crystal and vacuum respectively. Scale bars represent integrated intensity at each pixel position.

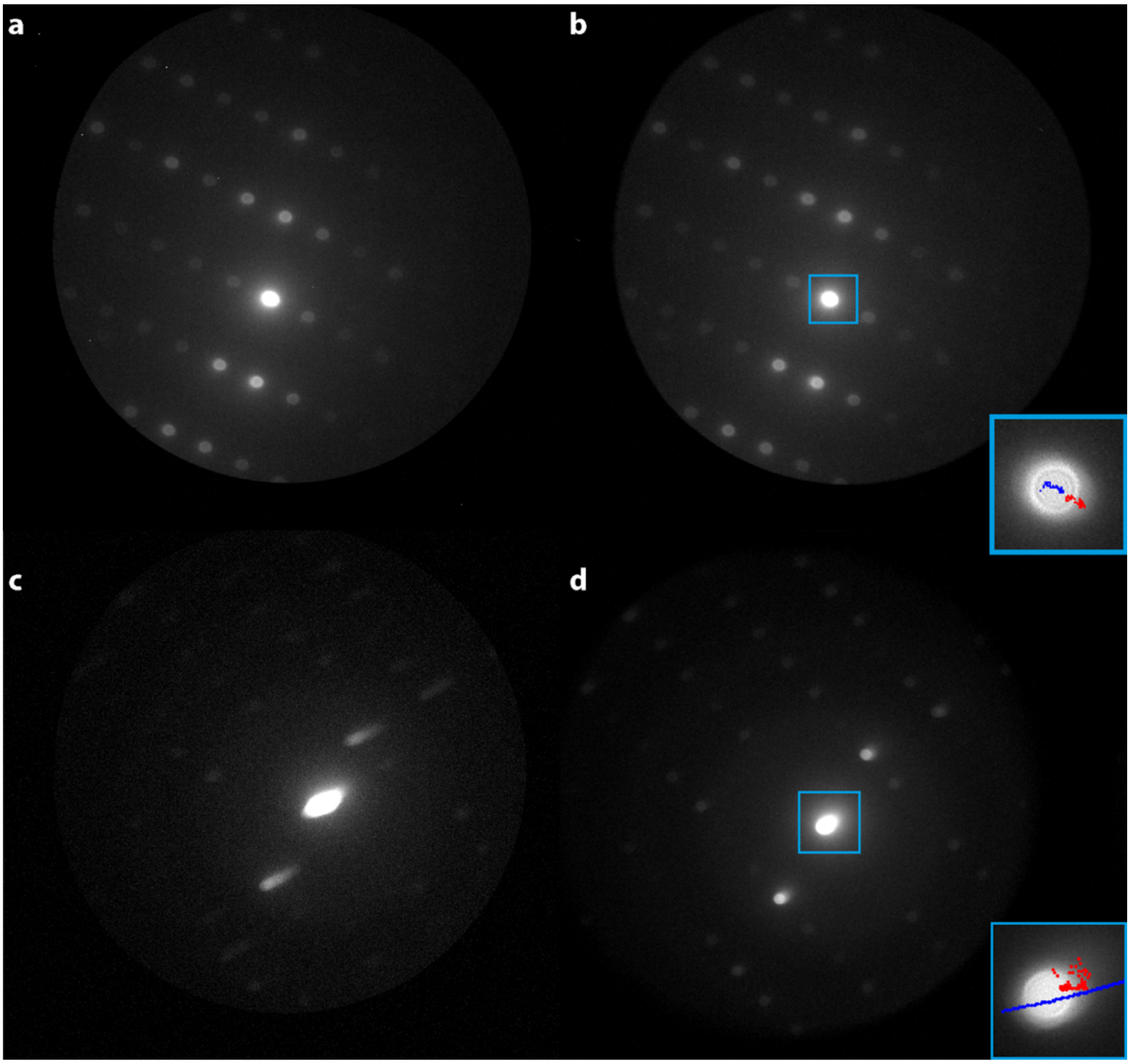


Supplementary Figure 6. **Beam shift correction in 4DSTEM datasets.** Mean diffraction of a 4DSTEM datasets showing minimal beam shift due to tilting of the probe during low magnification scanning acquisition both before (**a**) and after (**b**) shift correction. The amount of drift in first and second scan directions is represented as blue and red circles (inset). (**c**) Dataset showing large beam shift before correction. (**d**) Reduction in beam shift visible after correction. The amount of drift in first and second scan directions is represented as blue and red circles (inset).

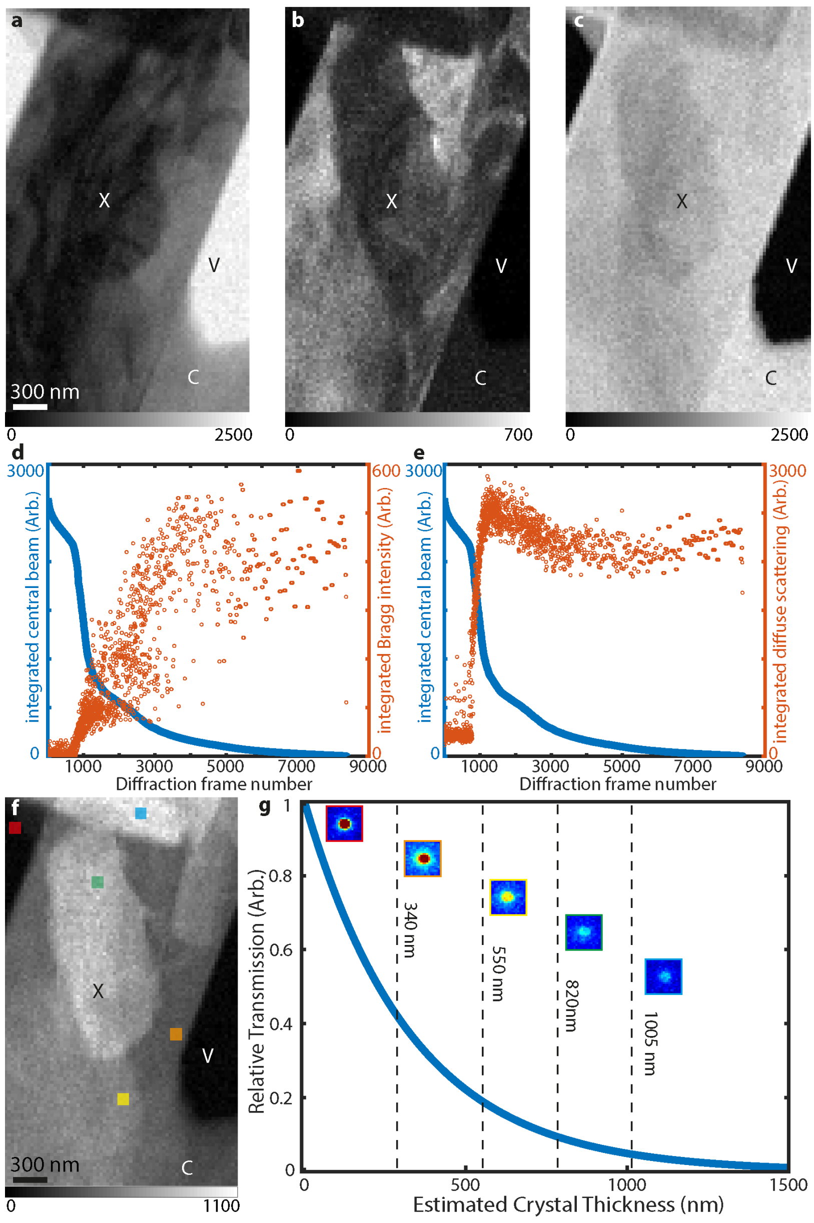


Supplementary Figure 7. **Estimation of crystal thickness from 4DSTEM patterns. (a - c)** Images reconstructed by integrating (**a**) the central beam, (**b**) all Bragg reflections or (**c**) the diffuse scattering within each 4DSTEM pattern. Colourbars represent integrated intensity. (**d**) Plot of integrated Bragg peaks as a function of central beam intensity. (**e**) Plot of diffuse background intensity as a function of central beam intensity. (**f**) Reconstructed image of crystal thickness estimated by combining information in (**a**) and (**b**), colourbar represents thickness in nm. (**g**) Plot of focused, central beam transmission as a function of sample thickness in a 4DSTEM scan. Inset images show the primary beam at the positions indicated by correspondingly coloured squares on the thickness map (**f**). All images are on the same intensity scale where red indicates high transmission and blue, low. C, X and V represent areas over carbon support, peptide crystal and vacuum respectively.

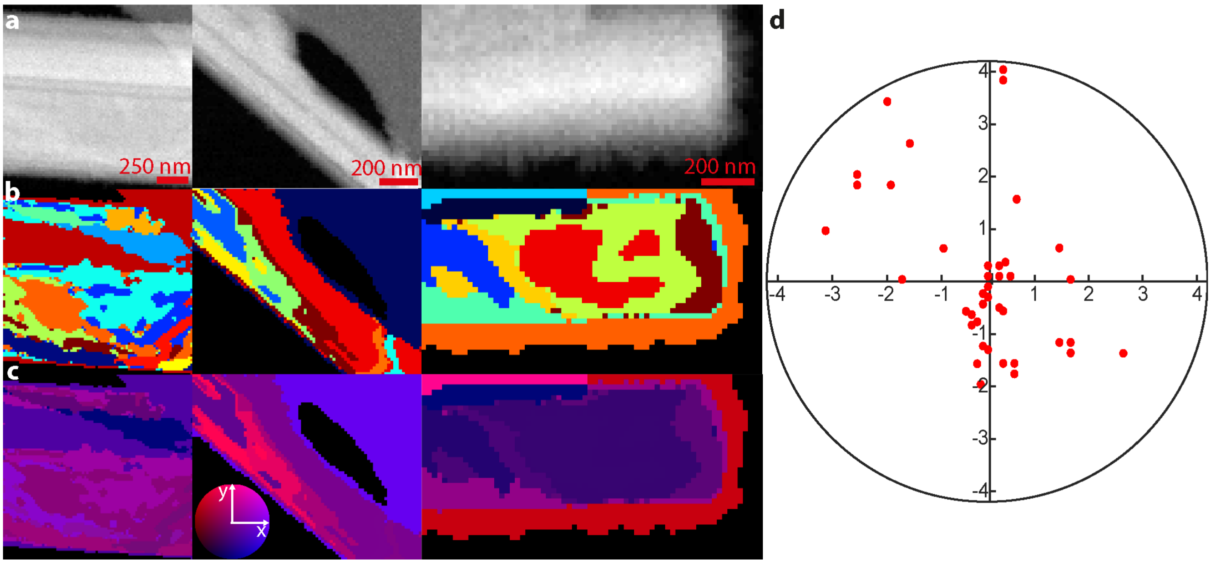


Supplementary Figure 8. **Representative HAADF images and orientation maps acquired from peptide nanocrystals by 4DSTEM.** (**a**) HAADF images of QYNNQNNFV crystals. (**b**) Maps of diffraction localization reconstructed from 4DSTEM scans of the same crystals as (**a**). (**c**) Maps recoloured after orientation assignment. The colour wheel (inset) demonstrates the relative orientation away from the mean in x and y-tilt, with a maximum deviation of 4 degrees. (**d**) Scatter plot of angular deviation from mean crystal orientation identified across QYNNQNNFV crystals analysed by 4DSTEM. Axes represent angular offset in degrees for the x and y-tilt directions respectively.

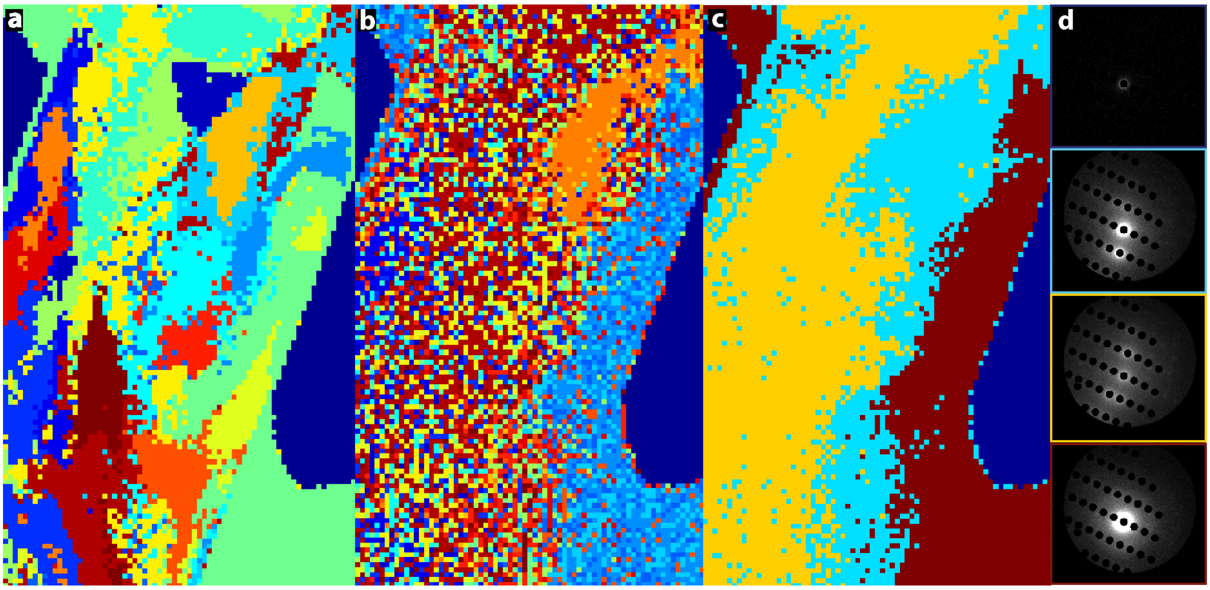


Supplementary Figure 9. **Influence of signal outside Bragg reflections on pattern classification and diffraction mapping.** (**a**) Map of diffraction clusters obtained from the nanocrystal seen in Fig. 2 in which 20 clusters were identified using the complete diffraction pattern (disregarding the primary beam). (**b**) Cluster map with 20 clusters calculated by excluding Bragg reflections - using only the diffuse scattering between Bragg peaks. (**c**) Cluster map calculated as in (**b**) but limited to 4 clusters. When the number of clusters is restricted, the map more closely follows the thickness map of the crystal. (**d**) Cluster averages for each of the 4 clusters shown in (**c**) with Bragg peaks removed.

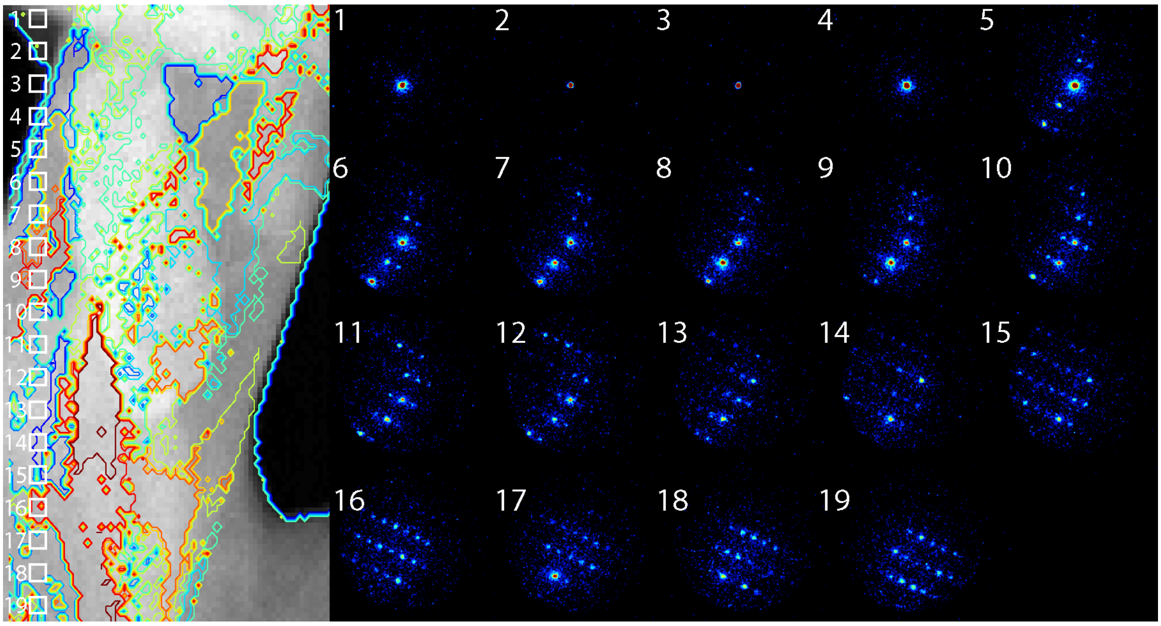


Supplementary Figure 10. **Smooth variation of diffraction as a function of spatial location within a nanocrystal.** (**Left**) HAADF image of a QYNNQNFV nanocrystal with contour lines outlining the clusters presented in Fig. 2(**b**). The numbered boxes indicate the region of the crystal that diffraction patterns (**right**) were acquired from. These patterns represent a 3x3 spatial average of diffraction data within the boxed region.

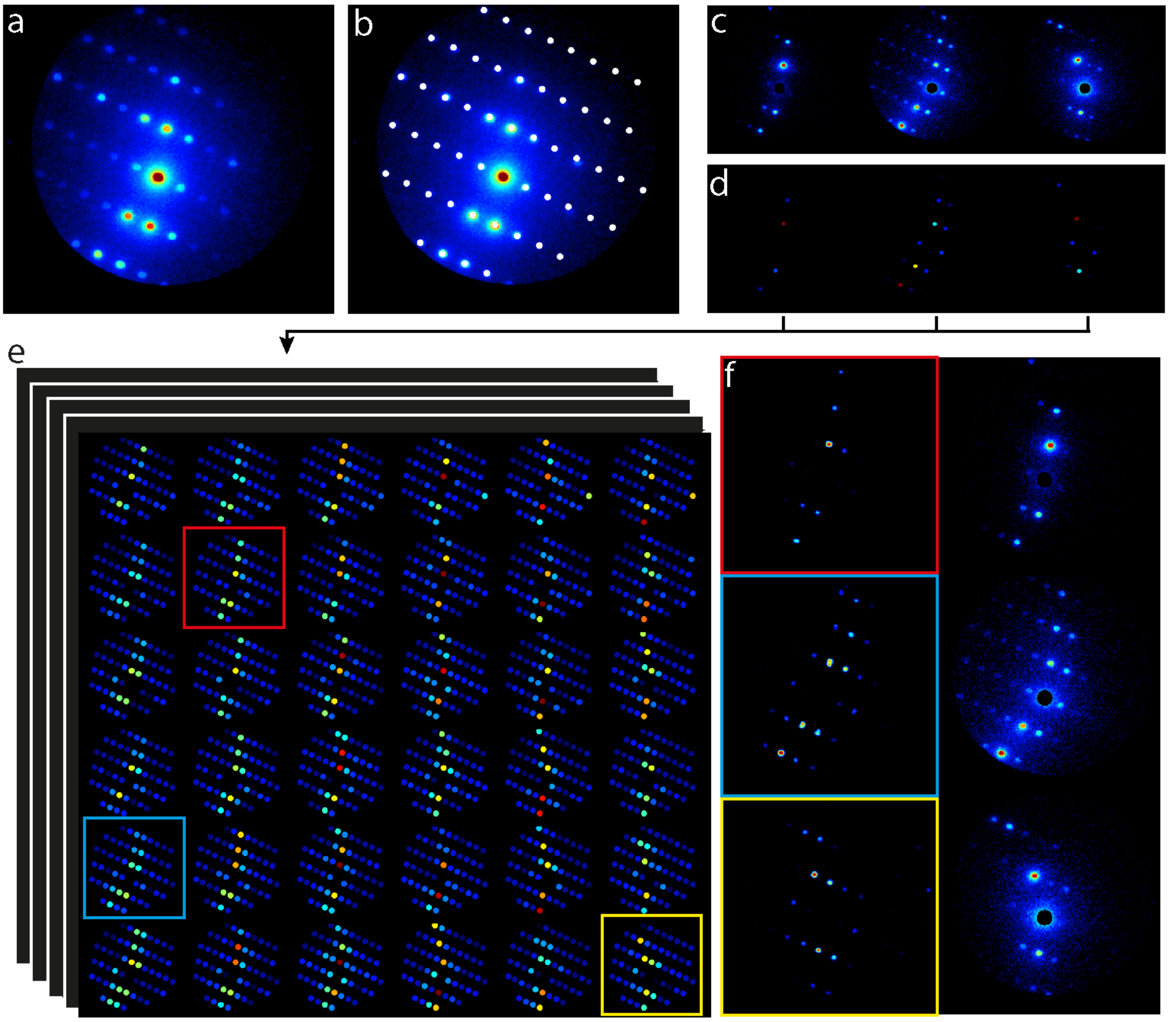


Supplementary Figure 11. **Orientation assignment of nanodiffraction cluster averages by library matching.** (**a**) Summed intensity of all patterns within a 4DSTEM scan. (**b**) Identification of all possible Bragg peaks that could be excited within the bounds of the HAADF detector. (**c**) Average patterns from individual crystalline regions. (**d**) Reduction of patterns to a list of intensities (represented by solid circles) and their respective *kxky* locations. (**e**) Library of simulated NBED patterns, best matches are highlighted by coloured boxes. (**f**) Results of library matching with best matched simulated patterns (left) and the corresponding cluster averages (right).

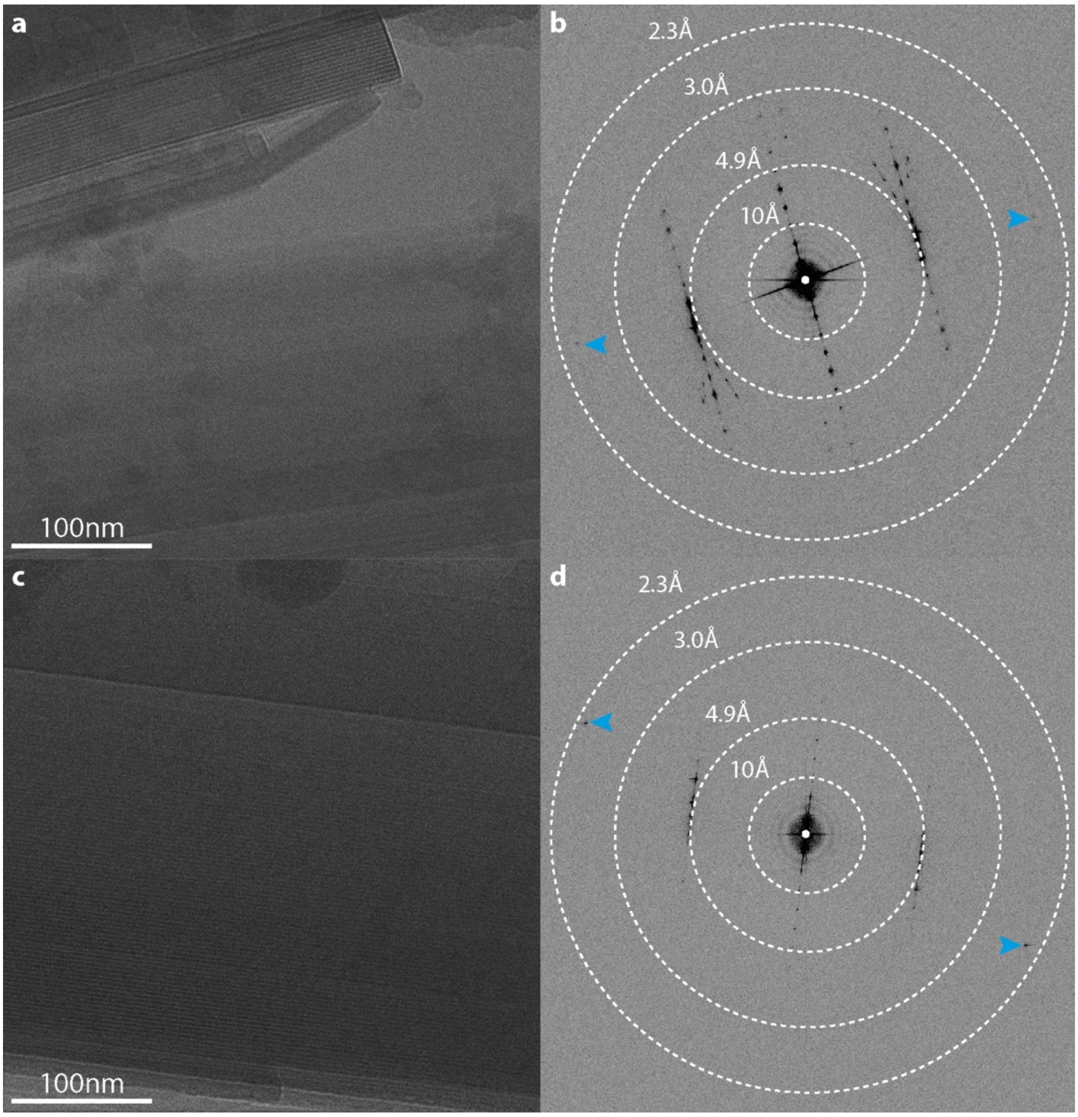


Supplementary Figure 12. **Crystal lattices observed in HRTEM images of crystal clusters and single nanocrystals.** (**a**) High resolution TEM image as shown in Fig. 3a. (**b**) Fourier transform of (**a**) with resolution rings. Blue arrows indicate highest resolution Bragg peaks at just less than 2.3Å resolution (**c**) High resolution TEM image as shown in Fig. 3d. (**d**) Fourier transform of (**c**) with resolution rings. Blue arrows indicate highest resolution Bragg peaks at just less than 2.3Å resolution.

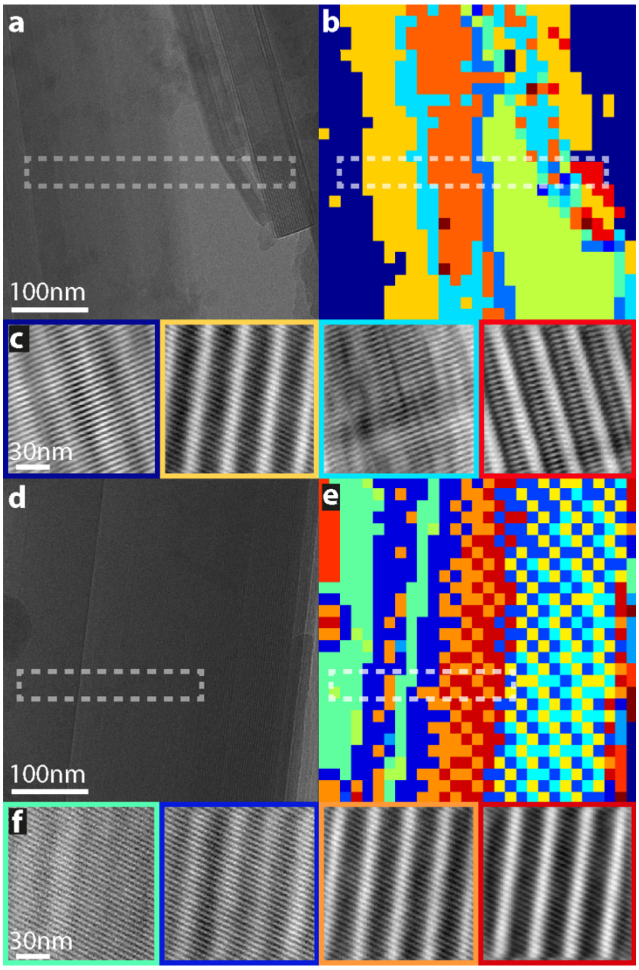


Supplementary Figure 13. **Mapping of crystalline regions within peptide nanocrystals by HRTEM.** (**a**) HRTEM image of a cluster of QYNNQNNFV nanocrystals. (**b**) Map of lattice changes present in the region imaged in (**a**). (**c**) Fourier filtered averages of regions within the dashed white box shown in (**a**) and (**b**). **(d)** HRTEM image of a single QYNNQNNFV nanocrystal. (**e**) Map of lattice changes present in the single crystal visible in (**a**). (**f**) Fourier filtered averages of regions in the dashed white box in (**d**) and (**e**).

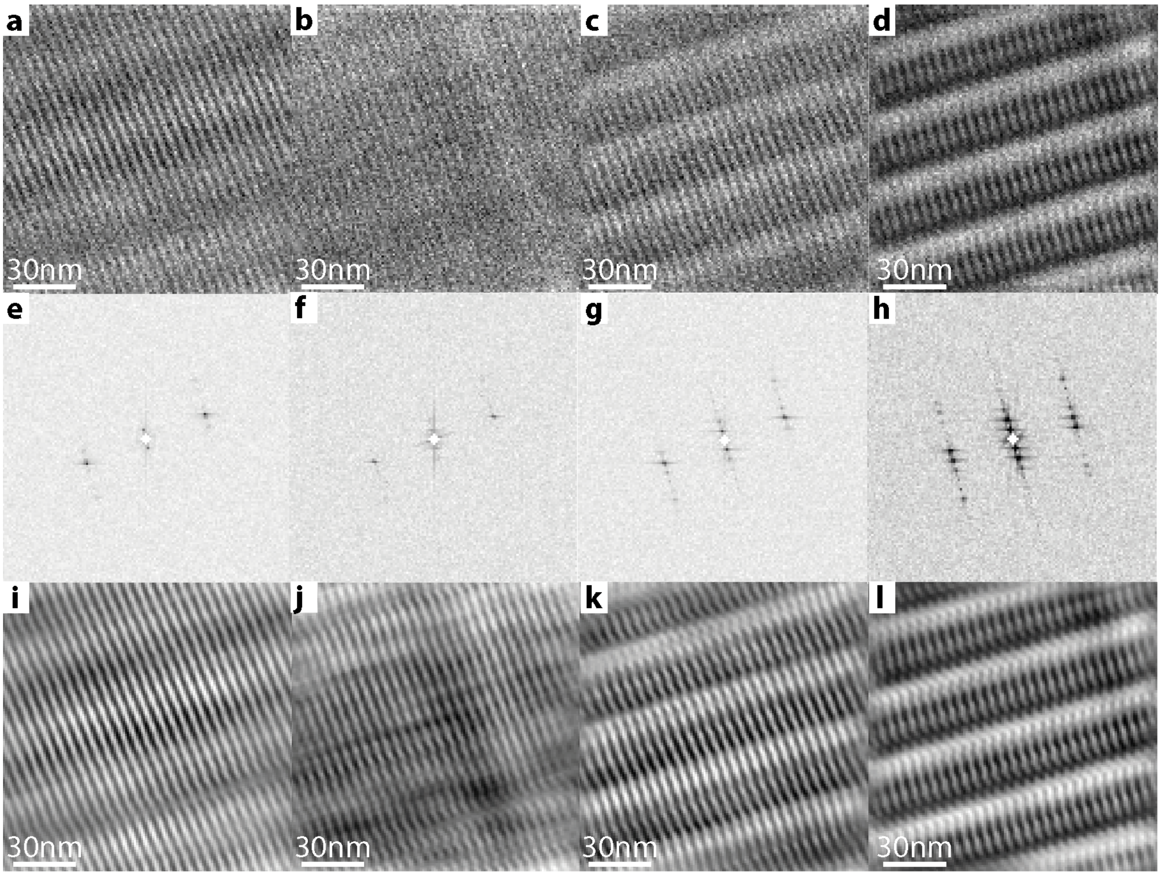


Supplementary Figure 14. **Fourier filtering of HRTEM cluster averages**. (**a - d**) Raw cluster averages before filtering from the image in Fig. 3a. (**e - h**) Fourier transforms of (**a - d**) showing the presence of Bragg peaks within the image transforms. (**i - l**) Cluster averages after Fourier filtering (as Fig. 3c).

## Supplementary Tables

Supplementary Table 1. **Experimental parameters for measurement of electron diffraction of ordered molecular assemblies**

| | Panova et al (2016) | This experiment | MicroED |
|---|---|---|---|
| Instrument | FEI Titan | TEAM I | FEI Tecnai F20 |
| Detector | Gatan Orius | Gatan K2 IS | TVIPS F416 |
| Temperature (K) | 77 | 77 | 77 |
| Accelerating voltage (keV) | 200 | 300 | 200 |
| Flux ($e^-$/s) | $3.2x10^7$ | $1.5x10^6$ | $2.4x10^5$ |
| Probe size (nm at FWHM) | 7 | 6 | 1000 (SA aperture) |
| Probe convergence angle (mrad) | 0.51 | 0.5 | near parallel |
| Estimated dose ($e^-$/Å$^2$) | 600 | 1 | 0.01 |
| Dwell time (ms) | 70 | 2.5 | 3000 |
| Step size (nm) | 20 – 40 | 15 - 20 | N/A |
